# Identifying risk groups of infectious spondylitis in patients with end-stage renal disease under hemodialysis: a propensity score-matched case-control study

**DOI:** 10.1186/s12882-019-1504-x

**Published:** 2019-08-16

**Authors:** Kun-Lin Lu, Wen-Hung Huang, Yueh-An Lu, Chan-Yu Lin, Hsin-Hsu Wu, Ching-Wei Hsu, Cheng-Hao Weng, Chao-Yi Wu, I-Wen Wu, Meng-Yu Wu, Tzung-Hai Yen, Huang-Yu Yang

**Affiliations:** 1 Chang Gung Memorial Hospital, Taoyuan, Taiwan; 2 Department of Nephrology, Chang Gung Memorial Hospital, College of Medicine, Chang Gung University, No. 5, Fusing St., Gueishan District, Taoyuan City, 333 Taiwan; 3 Division of Allergy, Asthma, and Rheumatology, Department of Pediatrics, Chang Gung Memorial Hospital, Chang Gung University College of Medicine, Taoyuan, Taiwan; 4 Department of Nephrology, Chang Gung Memorial Hospital, College of Medicine, Chang Gung University, Keelung, Taiwan; 5 Department of Thoracic and Cardiovascular Surgery, Chang Gung Memorial Hospital, College of Medicine, Chang Gung University, Taoyuan, Taiwan; 6 Bloomberg School of Public Health, Johns Hopkins University, Baltimore, MD USA

**Keywords:** Infectious spondylitis, Vertebral osteomyelitis, End-stage renal disease, Hemodialysis, Hematogenous infection, Albumin, Malnutrition, Red blood cell volume distribution width, Recent access operation, Propensity score-matched case-control study

## Abstract

**Background:**

Patients with end-stage renal disease (ESRD) under hemodialysis (HD) are at greater risks of infectious spondylitis (IS), but there is no reliable predictor that facilitate early detection of this relatively rare and insidious disease.

**Methods:**

A retrospective review of the medical records from patients with ESRD under HD over a 12-year period was performed at a tertiary teaching hospital, and those with a first-time diagnosis of IS were identified. A 1:4 propensity score-matched case-control study was carried out, and baseline characteristics, underlying diseases, and laboratory data were compared between the study group and the control group, one month before the date of diagnosis or the index date respectively.

**Results:**

A total of 16 patients with IS were compared with 64 controls. After adjustment, recent access operation (odds ratio [OR], 13.27; 95% confidence interval [CI], 3.53 to 49.91; *p* <  0.001), degenerative spinal disease (OR, 12.87; 95% CI, 1.89 to 87.41; *p* = 0.009), HD through a tunneled cuffed catheter (OR, 6.75; 95% CI, 1.74 to 26.14; *p* = 0.006), low serum levels of hemoglobin, albumin, as well as high levels of red blood cell volume distribution width (RDW), alkaline phosphatase (ALP), and high sensitivity C-reactive protein were significant predictors for a IS diagnosis one month later. Receiver operating characteristic curves for hemoglobin, RDW, ALP, and albumin all showed good discrimination. The further multivariate models identified both high serum ALP levels and low serum RDW levels following a recent access intervention in patients with relatively short HD vintages may be indicative of the development of IS.

**Conclusion:**

Patients under HD with relatively short HD vintages showing either elevated ALP levels or low RDW levels following a recent access intervention should prompt clinical awareness about IS for timely diagnosis.

**Electronic supplementary material:**

The online version of this article (10.1186/s12882-019-1504-x) contains supplementary material, which is available to authorized users.

## Background

Infectious spondylitis (IS), also known as pyogenic spondylitis, is a rare but life-threatening condition [1]. Clinical entities of IS include vertebral osteomyelitis, septic discitis, and epidural abscess. IS most commonly arises from hematogenous spread of bacteria, and often involves adjacent vertebrae and the corresponding intervertebral disks. IS is a disease affecting elderlies in their fifth decade of life with increasing incidence every decade thereafter, and occurs 1.5 times more frequently in male [2, 3]. Because most IS cases were believed to be predisposed by bacteremia, the reported risk factors were statuses related to suboptimal immunity, including old age, diabetes mellitus (DM), chronic kidney disease (CKD) and other immunocompromising conditions [4, 5]. In addition, any direct route into the bloodstream raises the risk of systemic bacterial infection. These risk factors synergistically contribute to the morbidity and mortality of patients with end-stage renal disease (ESRD). For instance, it has been reported that the annual mortality due to sepsis was approximately 100 to 300 fold higher in dialysis patients as compared to that in the general population [6]. Therefore, patients with ESRD under hemodialysis (HD) are under constant risk of developing IS and require clinical attention.

To diagnose IS in patients with ESRD under HD, clinical, radiological, and laboratory evaluations are often required. Early identification of IS to prompt initiation of therapy is vital in ensuring successful treatment and preventing morbidity. Because of the insidious onset of IS symptoms and the fact that most of the specific changes in imaging tests require two to eight weeks after the onset of the infection, the diagnosis may be delayed and lead to serious complications including irreversible neurological deficits and death [7]. Thanks to the high sensitivities of serum markers such as C-reactive protein (CRP) and erythrocyte sedimentation rate (ESR), they could serve as screening tests prior to magnetic resonance imaging (MRI), the gold standard to diagnose and evaluate the extent of IS [1]. However, whether certain serum markers provide a further predictive potential for a future IS event in these patients remains to be explored.

Our aim was to perform a 1:4 propensity score-matched case-control study among HD patients to identify the differences in baseline characteristics and potential serum markers between the patients who developed IS one month later and those who did not. Because the differences in age, gender, and DM status may affect blood tests results and clinical outcomes, we matched on these important confounding factors and adjusted them in our models (as presented below).

## Methods

### Patient selection and matching process

This study retrospectively reviewed the electronic medical records and the HD records from the HD center of Chang Gung Memorial Hospital, Linko, a tertiary medical center in Taiwan. The HD center has 278 dialysis beds and serves 1550 patients monthly. Patients who received maintenance HD and had complete reports of monthly blood investigations in the HD center from 1 July, 2006 to 30 June, 2015 were identified. Patients who were over 20 years old and had been diagnosed with the first-time IS during the study period were classified as the study group.

**Fig. 1:**
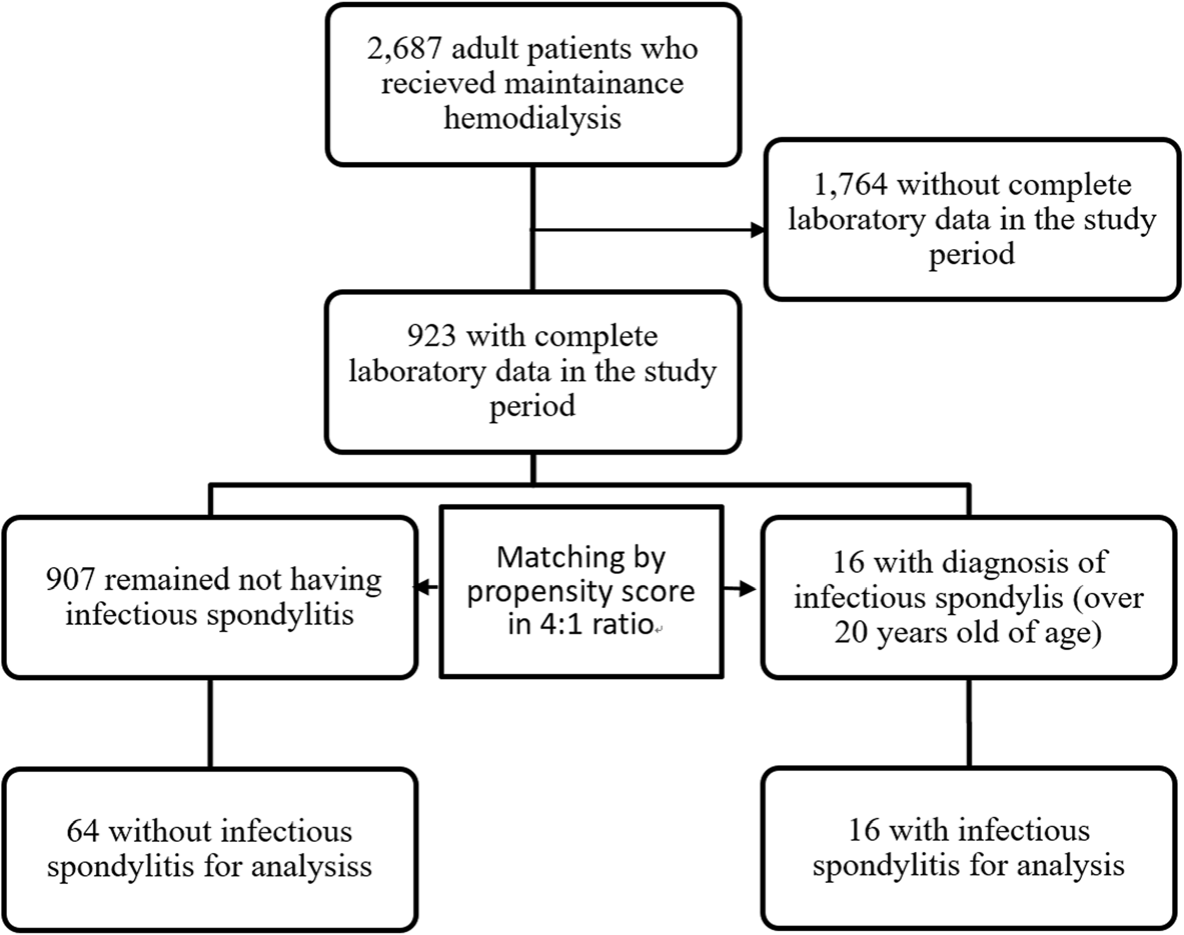
Flowchart of study profile

The method of propensity score matching (PSM) was applied to select the control group from patients without IS in the HD center. We assigned February 2011 as the index month for the comprehensive laboratory results collected. We carried out a PSM to match each of the IS cases with 4 controls by age at index month (± 2 years), exact gender, and exact status of DM. We further included HD vintage as an additional matching variable to perform the second propensity score matching model. The 4 controls for each IS case were randomly selected if there was no difference in these factors between eligible controls. All data was anonymized. The study protocol was approved by the Institution Review Board of Chang Gung Memorial Hospital, Linko. The flowchart of the study profile was presented in Fig. 1 below.

### Baseline covariates

Patient’s baseline characteristics and other comorbidities were retrieved from chart review. The characteristics that uniquely associated with ESRD were also documented from the HD records: the primary cause of ESRD, HD vintage (length of time on HD), patient’s HD access and if they had received any access operation within 6 months. The study recorded the results of routine serum investigations on the date nearest to the time point of one month before the diagnosis of IS of the study group and in the index month of the control group. These routine serum laboratory measurements were obtained in HD center monthly since the initiation of HD, including leukocyte count, hemoglobin, red blood cell volume distribution width (RDW), platelet count, albumin, alanine transaminase (ALT), alkaline phosphatase (ALP), blood urea nitrogen (BUN), creatinine, potassium, calcium, inorganic phosphorus, glucose Ante Cibum (AC), high sensitive C-reactive protein (hsCRP) and ferritin. In addition, due to the commonly delayed diagnosis of IS, we also collected data of these serum investigations on the date nearest to the time point of three months before the diagnosis of IS for comparisons.

### Statistical analyses

The standardized mean difference (SMD) of the continuous matching variable and age between the cases and the controls was calculated, with a cutoff level of 0.1 to signify an adequate balance of the matching. Shapiro-Wilk test was applied to determine the distribution of continuous variables. Skewed data, including levels of RDW, ALT, ALP, albumin, glucose, hsCRP, Ferritin, and HD vintage were analyzed by the Mann-Whitney test. For other data with normal distribution, independent t-test or Pearson’s chi-squared test were applied for linear or categorical variables respectively. We further tested each of the significant factors with binary logistic regression adjusted for age, gender, and DM status and reported the adjusted odds ratios and 95% confident intervals (CIs) for incident IS. For the covariant “recent access operation”, we also performed a logistic regression model with inverse propensity score weighting. The propensity scores for recent access operation were calculated based on age, gender, and DM status. Receiver operating characteristic (ROC) curves and cutoff values for predicting future IS diagnosis were identified for the significantly-related continuous variables after adjustment, including albumin, RDW, hemoglobin and ALP levels. Missing data (of hsCRP) was replaced using multiple imputations before analysis.

We further tested multivariate binary logistic regression analysis adjusting for age, gender, and DM status and with forward selection of the significant variables to estimate the odds ratios and 95% CIs for incident IS within the model. Variables within the same regression model had also been checked for collinearity and interaction. After identifying the most significant combination related to IS, a multivariate ROC curve was carried out based on the new variable “probability” calculated from this model.

All reported *p* values are two-sided, and *p* values less than 0.05 were considered statistically significant. All statistical analyses were performed using Immersed Boundary Method (IBM) Statistical Package for the Social Sciences (SPSS) Statistics, version 22.

## Results

The study group contained 16 patients aged 20 years or older, who received regular HD and had been diagnosed with IS from 1 July 2006 to 30 June 2015. For each patient in the study group, 4 controls were matched by propensity score by age, gender, and status of DM. This matching was confirmed to be adequately balanced by calculating the level of SMDs of age, which was 0.0258.

The baseline characteristics according to the grouping are shown in Table 1 below. Patients between the study and control group had no statistical difference in age, gender, DM, hypertension, coronary artery diseases, heart failure, cerebral vascular diseases, liver cirrhosis, malignancy, and history of spine operation. Patients with IS had higher prevalence of immunosuppression status (6.3% vs. 0%, *p* = 0.04) and degenerative spinal disease (87.5% vs. 51.6%, *p* = 0.01). Patients in the study group had a shorter HD vintage (4.65 ± 4.36 vs. 13.44 ± 7.30, *p* <  0.001), a higher possibility of receiving HD through tunneled cuffed catheter (43.8% vs. 12.5%, *p* <  0.01) rather than arteriovenous fistula (37.5% vs. 68.8%, *p* = 0.02), and a higher likelihood of receiving access operation within 6 months (62.5% vs. 12.5%, *p* <  0.001). Patients in both groups had no significant difference in their primary cause of ESRD.

**Table 1:** Baseline characteristics and clinical features of hemodialysis patients with and without infectious spondylitis (model 1)

Variables	Patients with infectious spondylitis (*n* = 16)	Patients without infectious spondylitis (*n* = 64)	*p* value
Age, mean ± SD, year	60.9 ± 11.9	60.6 ± 11.3	0.93
Male Gender, *n* (%)	7 (43.8%)	28 (43.8%)	1.00
Diabetes mellitus, *n* (%)	6 (37.5%)	24 (37.5%)	1.00
Hypertension, *n* (%)	8 (50%)	46 (71.9%)	0.10
Coronary artery diseases, *n* (%)	3 (18.8%)	5 (7.8%)	0.19
Heart failure, *n* (%)	1 (6.3%)	4 (6.3%)	1.00
Cerebral vascular diseases, *n* (%)	1 (6.3%)	7 (10.9%)	0.58
Liver cirrhosis, *n* (%)	2 (12.5%)	2 (3.1%)	0.12
Malignancy, *n* (%)	1 (6.3%)	7 (10.9%)	0.58
Immunosuppression status, *n* (%)	1 (6.3%)	0 (0%)	0.04
Degenerative spinal disease, *n* (%)	14 (87.5%)	33 (51.6%)	0.009
History of spinal operation, *n* (%)	1 (6.3%)	3 (4.7%)	0.80
Primary cause of ESRD			0.18
Chronic Glomerulonephritis	7 (43.8%)	34 (53.1%)	
Diabetic nephropathy	6 (37.5%)	24 (37.5%)	
Polycystic kidney disease	2 (12.5%)	3 (4.7%)	
Malignant hypertension	1 (6.3%)	0 (0%)	
Others	0 (0%)	3 (4.7%)	
HD vintage (years)	4.65 ± 4.36	13.44 ± 7.30	< 0.001
HD access			
Arteriovenous fistula	6 (37.5%)	44 (68.8%)	0.021
Arteriovenous graft	3 (18.8%)	12 (18.8%)	1.00
Tunneled cuffed catheter	7 (43.8%)	8 (12.5%)	< 0.001
Access operation within recent 6 months	10 (62.5%)	8 (12.5%)	< 0.001
Laboratory data (blood sample)			
Leukocyte count, 1000/μL	7.53 ± 2.2	6.62 ± 1.7	0.08
Hemoglobin, g/dL	8.86 ± 1.3	10.2 ± 1.6	< 0.001
RDW, %	16.3 ± 2.2	14.5 ± 1.4	< 0.001
Platelet Count, 1000/μL	181 ± 76	192 ± 54	0.48
BUN, mg/dL	68.2 ± 27.8	67.9 ± 17.3	0.97
Creatinine, mg/dL	9.10 ± 3.15	10.4 ± 2.3	0.06
Potassium, mEq/L	4.60 ± 0.74	5.00 ± 0.76	0.06
Calcium, mg/dL	9.54 ± 1.08	9.76 ± 0.91	0.42
Inorganic phosphorus, mg/dL	4.98 ± 1.99	5.11 ± 1.49	0.77
ALT, U/L	18.2 ± 13.2	15.5 ± 8.5	0.32
ALP, U/L	131.2 ± 84.5	83.4 ± 38.8	0.001
Albumin, g/dL	3.35 ± 0.65	3.90 ± 0.38	0.001
Glucose AC, mg/dL	140 ± 91	129 ± 88	0.67
hsCRP, mg/L	33.1 ± 43.6	12.4 ± 23.9	0.004
Ferritin, ng/mL	475.7 ± 513.2	335.4 ± 312.7	0.31

*ESRD* end stage renal disease, *HD* hemodialysis, *RDW* red blood cell volume distribution width, *ALT* alanine transaminase, *ALP* alkaline phosphatase, *BUN* blood urea nitrogen, *AC* ante cibum, *hsCRP* high sensitivity C- reactive protein

The blood investigation results of the study group one month before the diagnosis of IS showed significantly lower hemoglobin level (8.86 ± 1.3 vs. 10.2 ± 1.6, *p* <  0.001), higher RDW (16.3 ± 2.2 vs. 14.5 ± 1.4, *p* = 0.001), higher ALP (131.2 ± 84.5 vs. 83.4 ± 38.8, *p* = 0.001), lower albumin (3.35 ± 0.65 vs. 3.90 ± 0.38, *p* = 0.001), and higher hsCRP (33.1 ± 43.6 vs. 12.4 ± 23.9, *p* = 0.004) compared to the control group. On the other hand, leukocyte count, platelet, BUN, creatinine, potassium, calcium, phosphorous, ALT, glucose, and ferritin level were not significantly different between the two groups.

Regarding HD vintage, we found a significant association between it and a recent access operation (*p* = 0.032). In addition, we also identified positive relationships between HD vintage and both serum levels of albumin and hemoglobin (*p* = 0.003 and 0.042, respectively). To clarify its impact on our findings, we further performed the second propensity score matching model adding HD vintage as a matching variable and showed overall similar results as demonstrated in Table 2, with the exception being there were less underlying disease of hypertension (50% vs. 79.7%, *p* = 0.02) in the IS group.

**Table 2:** Baseline characteristics and clinical features of hemodialysis patients with and without infectious spondylitis (model 2)

Variables	Patients with infectious spondylitis (*n* = 16)	Patients without infectious spondylitis (*n* = 64)	*p* value
Age, mean ± SD, year	60.9 ± 12.0	61.0 ± 11.5	0.98
Male Gender, *n* (%)	7 (43.8%)	22 (34.4%)	0.48
Diabetes mellitus, *n* (%)	6 (37.5%)	22 (34.4%)	0.82
Hypertension, *n* (%)	8 (50%)	51 (79.7%)	0.02
Coronary artery diseases, *n* (%)	3 (18.8%)	9 (14.1%)	0.64
Heart failure, *n* (%)	1 (6.3%)	10 (15.6%)	0.33
Cerebral vascular diseases, *n* (%)	1 (6.3%)	6 (9.4%)	0.69
Liver cirrhosis, *n* (%)	2 (12.5%)	4 (6.3%)	0.40
Malignancy, *n* (%)	1 (6.3%)	8 (12.5%)	0.48
Immunosuppression status, *n* (%)	1 (6.3%)	0 (0%)	0.04
Degenerative spinal disease, *n* (%)	14 (87.5%)	3 (4.7%)	< 0.001
History of spinal operation, *n* (%)	1 (6.3%)	3 (4.7%)	0.80
Primary cause of ESRD			0.73
Chronic Glomerulonephritis	7 (43.8%)	37 (57.8%)	
Diabetic nephropathy	6 (37.5%)	15 (23.4%)	
Polycystic kidney disease	2 (12.5%)	9 (14.1%)	
Malignant hypertension	1 (6.3%)	2 (3.1%)	
Others	0 (0%)	1 (1.6%)	
HD vintage (years)	4.65 ± 4.36	5.03 ± 3.59	0.42
HD access			
Arteriovenous fistula	6 (37.5%)	53 (82.8%)	< 0.001
Arteriovenous graft	3 (18.8%)	7 (10.9%)	0.40
Tunneled cuffed catheter	7 (43.8%)	4 (6.3%)	< 0.001
Access operation within recent 6 months	10 (62.5%)	9 (14.1%)	< 0.001
Laboratory data (blood sample)			
Leukocyte count, 1000/μL	7.53 ± 2.2	6.57 ± 2.2	0.13
Hemoglobin, g/dL	8.86 ± 1.3	9.82 ± 1.3	0.012
RDW, %	16.3 ± 2.2	14.2 ± 1.4	< 0.001
Platelet Count, 1000/μL	181 ± 76	198 ± 68	0.37
BUN, mg/dL	68.2 ± 27.8	65.4 ± 18.7	0.62
Creatinine, mg/dL	9.10 ± 3.15	10.1 ± 2.4	0.18
Potassium, mEq/L	4.60 ± 0.74	4.79 ± 0.77	0.36
Calcium, mg/dL	9.54 ± 1.08	9.81 ± 0.95	0.34
Inorganic phosphorus, mg/dL	4.98 ± 1.99	5.07 ± 1.52	0.84
ALT, U/L	18.2 ± 13.2	15.3 ± 11.1	0.20
ALP, U/L	139.9 ± 79.7	84.5 ± 45.0	0.001
Albumin, g/dL	3.35 ± 0.65	3.97 ± 0.42	< 0.001
Glucose AC, mg/dL	149 ± 86	124 ± 70	0.07
hsCRP, mg/L	29.90 ± 47.02	8.18 ± 16.85	0.024
Ferritin, ng/mL	475.7 ± 513.2	419.9 ± 485.3	0.85

*ESRD* end stage renal disease, *HD* hemodialysis, *RDW* red blood cell volume distribution width, *ALT* alanine transaminase, ALP alkaline phosphatase, *BUN* blood urea nitrogen, *AC* ante cibum, *hsCRP* high sensitivity C- reactive protein

Variables that were significantly different in patient’s baseline characteristics and clinical features were adjusted by sex, gender and DM in order to identify the risk factors for IS in HD patients (as shown in Table 3). In the first matching model, the following variables remained significant after adjustment: underlying disease of degenerative spinal disease (odds ratio [OR] = 12.87, 95% confident intervals [CI] = 1.89–87.41; *p* = 0.009), HD vintage (OR = 0.64, 95%CI = 0.51–0.81; *p* <  0.001), HD through a tunneled cuffed catheter (OR = 6.75, 95%CI = 1.74–26.14; *p* = 0.006), access operation within recent 6 months (OR = 13.27, 95%CI = 3.53–49.91; *p* <  0.001), hemoglobin (OR = 0.47, 95% CI = 0.29–0.76; *p* = 0.002), RDW (OR = 2.01, 95% CI = 1.33–3.04; *p* = 0.001), ALP (OR = 1.02, 95% CI = 1.00–1.03; *p* = 0.008), albumin (OR = 0.09, 95% CI = 0.02–0.35; *p* = 0.001), hsCRP (OR = 1.02, 95%CI = 1.00–1.04; *p* = 0.046). The inverse propensity score weighting used to estimate the effect of recent access operation on IS consistently demonstrated a significant association of recent access operation with an IS in the near future (OR = 11.52, 95% CI = 3.20–41.50; *p* <  0.001).

**Table 3:** Adjusted odds ratios for significant variables related to infectious spondylitis (model 1)

Variables	OR (95% CI)^a^	*p* value
Immunosuppression status	7.649E+ 9	1.00
Degenerative spinal disease	12.87 (1.89–87.41)	0.009
HD vintage (years)	0.64 (0.51–0.81)	< 0.001
HD access		0.022
Arteriovenous fistula	1	–
Arteriovenous graft	1.827 (0.395–8.455)	0.44
Tunneled cuffed catheter	6.75 (1.74–26.14)	0.006
Access operation within recent 6 months	13.27 (3.53–49.91)	< 0.001
Hemoglobin	0.47 (0.29–0.76)	0.002
RDW	2.01 (1.33–3.04)	0.001
ALP	1.02 (1.00–1.03)	0.008
Albumin	0.09 (0.02–0.35)	0.001
hsCRP	1.02 (1.00–1.04)	0.046

^a^Adjusted for age, gender, and diabetes mellitus

*OR* odds ratio, *CI* confidence interval, *HD* hemodialysis, *RDW* red blood cell volume distribution width, *ALP* alkaline phosphatase, *hsCRP* high sensitivity C- reactive protein

When it comes to the second matching model, generally similar results were shown in Table 4. The main divergences being hsCRP no longer remained significantly different, while underlying disease of hypertension was significantly less in the IS group after adjustment.

**Table 4:** Adjusted odds ratios for significant variables related to infectious spondylitis (model 2)

Variables	OR (95% CI)^a^	*p* value
Immunosuppression status	9.17E+ 09	1.00
Degenerative spinal disease	1.90E+ 10	1.00
Hypertension	0.23 (0.068–0.783)	0.02
HD access		0.001
Arteriovenous fistula	1	–
Arteriovenous graft	4.15 (0.81–21.34)	0.09
Tunneled cuffed catheter	17.87 (3.69–86.49)	< 0.001
Access operation within recent 6 months	11.56 (3.18–42.01)	< 0.001
Hemoglobin	0.59 (0.38–0.90)	0.015
RDW	2.09 (1.40–3.12)	< 0.001
ALP	1.01 (1.00–1.02)	0.013
Albumin	0.11 (0.03–0.38)	0.001
hsCRP	1.02 (1.00–1.05)	0.07

^a^Adjusted for age, gender, and diabetes mellitus

*OR* odds ratio, CI confidence interval, *HD* hemodialysis, *RDW* red blood cell volume distribution width, *ALP* alkaline phosphatase, *hsCRP* high sensitivity C- reactive protein

**Fig. 2:**
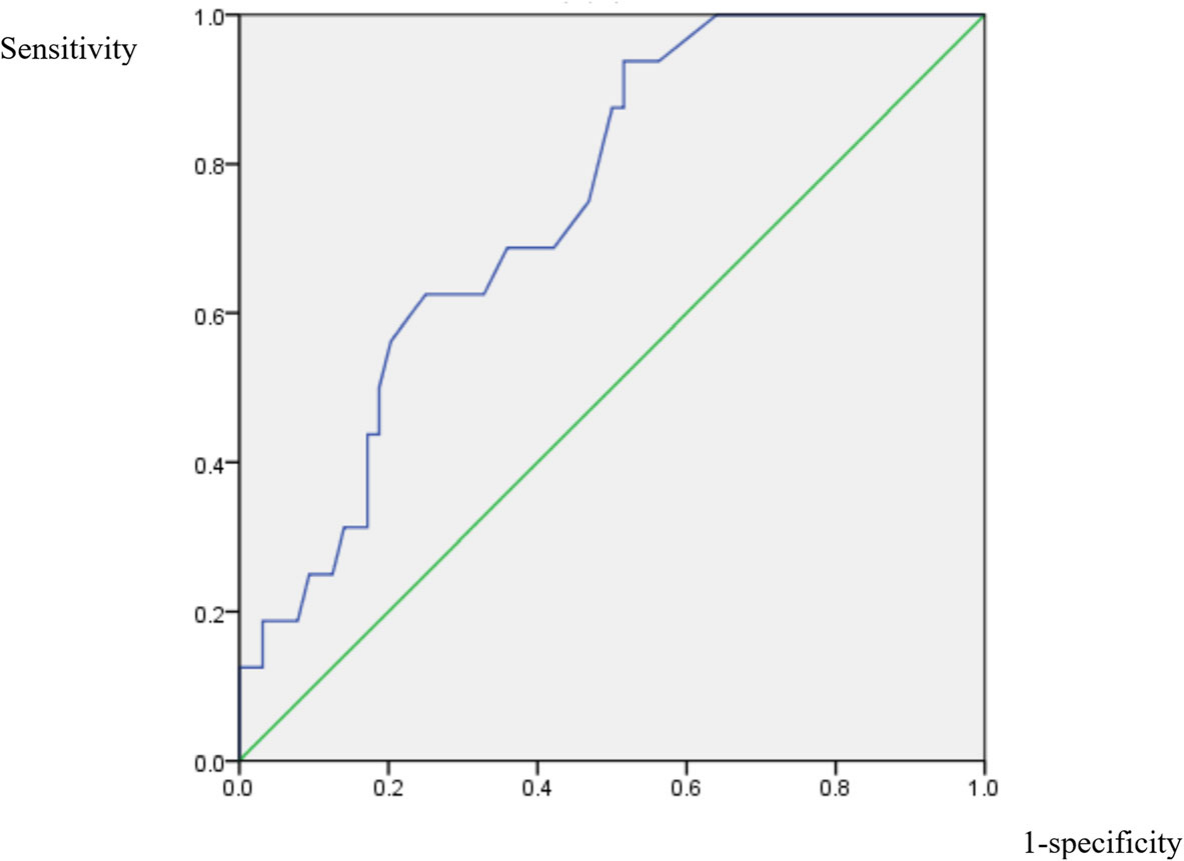
ROC curve of hemoglobin. Receiver operating characteristic curve for hemoglobin level to predict the diagnosis of infectious spondylitis

**Fig. 3:**
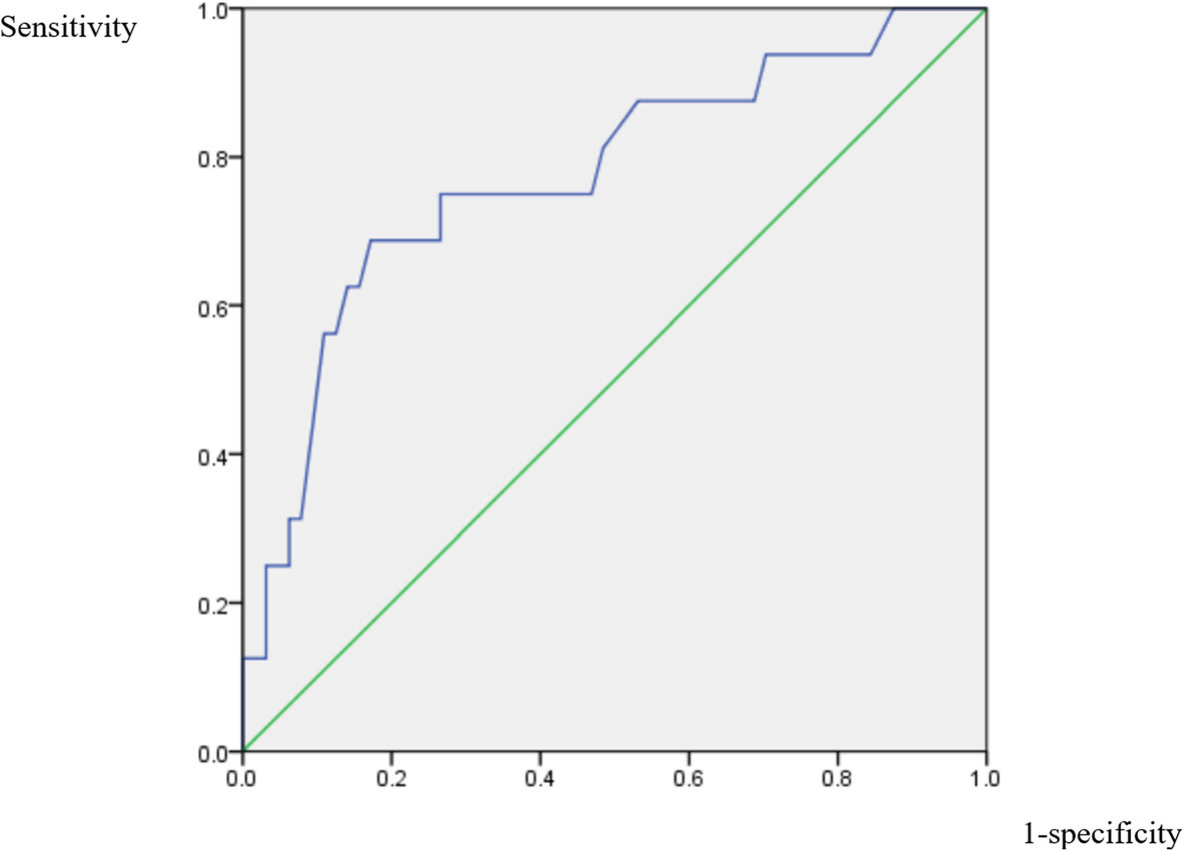
ROC curve of RDW. Receiver operating characteristic curve for the level of red blood cell volume distribution width to predict the diagnosis of infectious spondylitis

**Fig. 4:**
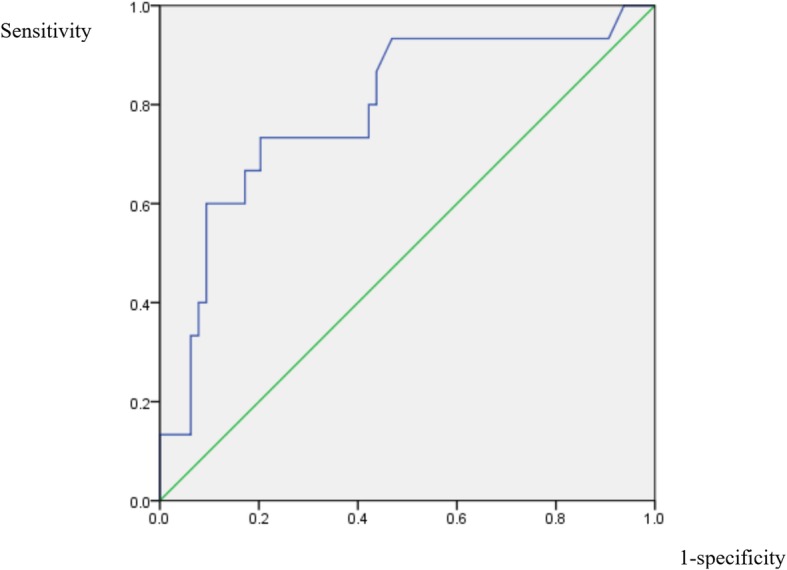
ROC curve of ALP. Receiver operating characteristic curve for the alkaline phosphatase level to predict the diagnosis of infectious spondylitis

**Fig. 5:**
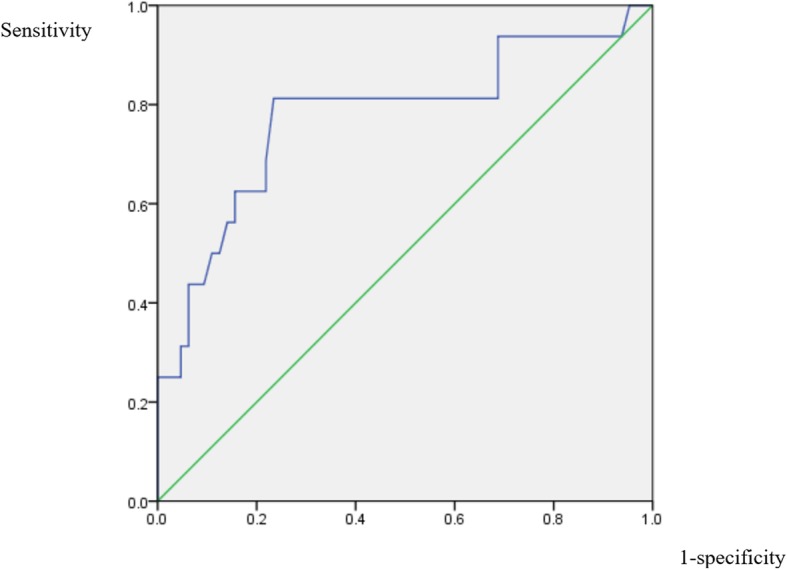
ROC curve of albumin. Receiver operating characteristic curve for the albumin level to predict the diagnosis of infectious spondylitis

The ROC curves for the variables that remained significantly-related after adjustment, hemoglobin, RDW, ALP, and albumin, were shown in Figs. 2, 3, 4 and 5. For hemoglobin, the area under the curve was 74.5% while 9.35 was the cutoff level as a risk factor for IS (sensitivity: 62.5%; specificity: 75.0%). For RDW, the area under the curve was 77.5% while 15.65% was the cutoff level for RDW to point at a possible diagnosis of IS (sensitivity: 68.8%; specificity: 82.8%). When it comes to that of ALP, the area under the curve was 79.0% and 103.5 was the cutoff level as a risk factor for IS (sensitivity: 73.3%; specificity: 79.7%). In the case of albumin, the area under the curve was 77.8% while 3.73 g/dL was the cutoff level for albumin to indicate an increased risk for IS (sensitivity: 81.2%; specificity: 76.6%).

We managed to evaluate adjusted predictive models for future IS by binary logistic regression analyses with forward selection of the significantly-related variables after adjustment. The first matching model demonstrates that the two selected factors following adjustment were HD vintage and ALP, as shown in Table 5. No collinearity was found between the chosen variables, and this multivariate ROC curve had an area under the curve of 92.3%.

**Table 5:** A forward-selected logistic regression model of variables associated with infectious spondylitis (model 1)

Explanatory variables	OR (95% CI)^a^	*p* value
HD vintage (years)	0.52 (0.34–0.78)	0.002
ALP	1.03 (1.01–1.05)	0.005

^a^Adjusted for age, gender, and diabetes mellitus

*OR* odds ratio, *CI* confidence interval, *HD* hemodialysis, *ALP* alkaline phosphatase

We also followed the same approach for the second matching model, turning out that the two selected factors following adjustment were RDW level and an access operation within recent 6 months, as shown in Table 6. No collinearity was found between the chosen variables, and the multivariate ROC curve had an area under the curve of 88.8%.

**Table 6:** A forward-selected logistic regression model of variables associated with infectious spondylitis (model 2)

Explanatory variables	OR (95% CI)^a^	*p* value
RDW	2.10 (1.40–3.38)	0.001
Access operation within recent 6 months	9.09 (1.94–42.70)	0.005

^a^Adjusted for age, gender, and diabetes mellitus

*OR* odds ratio, *CI* confidence interval, *RDW* red blood cell volume distribution width

In addition, we analyzed lab data collected around three months before the diagnosis of IS and compared them to those of one month before diagnosis. The findings remained consistent and no apparent difference was noted (Additional file 1: Table S1).

## Discussion

In the first propensity score-matched model matched by age, gender and DM status, we found that patients with the history of a degenerative spinal disease, shorter HD vintage, recent access operation, or HD through a tunneled cuffed catheter was significantly related to the development of IS after adjustment. Moreover, levels of laboratory data, including lower hemoglobin, lower serum albumin, higher RDW, higher ALP, and higher hsCRP were also significantly linked to IS after adjustment. Since we found HD vintage to be different between the two groups, which may be confounding, we built the second propensity score-matched model with the addition of HD vintage as a matching factor. Overall, consistent results were noted even after adjustment. In addition, we further compared these laboratory findings documented about three months before the diagnosis of IS to those collected one month before diagnosis in light of the insidious nature of IS, and consistent results were still noted. In order to ensure timely diagnosis of IS in clinically-suspected HD patients, we also identified optimal cutoff-levels of serum hemoglobin, RDW, ALP, and albumin. To our knowledge, this is the first study to show relationships between these variables and the odds of IS among HD patients.

The significant relationships linking recent access operation and tunneled cuffed catheter to increased odds of IS plausibly suggest an increased risk of catheter access infection due to transient bacteremia in these patients on HD [8]. When it comes to laboratory studies, lower hemoglobin and albumin levels have long been linked to inflammation and malnutrition. It is well-known that HD poses an increased risk of inflammatory syndromes in patients through both patient-related and HD-related factors [9]. This chronic inflammatory effect had been consistently reported to be related to decreased serum albumin level [10] and subsequently increased mortality [11]. In addition, malnutrition also increases the mortality in patients on HD [12, 13], likely through decreasing the number of T cells and impairing the production of cytokines [14]. On the other hand, the associations of both increased RDW and increased ALP with the mortality of HD patients have both been reported [15–17]. Elevated RDW may reflect multiple underlying conditions, including ineffective erythropoiesis, malnutrition, increased oxidative stress, and endothelial dysfunction [18]. Although the increase in ALP has been reported as a reliable marker for the severity of the high-turnover osteodystrophy in patients on HD [19], and is associated with renal osteodystrophy and vitamin D deficiency [20], this is an independent risk factor for altered immunity and inflammation [21]. As for hsCRP, both of its indicative role of inflammatory status and its prognostic part in HD patients have been well-recognized [22–24]. Our result demonstrated that elevated levels of these laboratory findings should raise clinical concerns for the inflammatory process linking to an increased risk for IS in addition to the HD status.

After clarifying the adjusted odds ratios for each variables for IS, we further managed to generate a multivariable model with adjustment to look into the combinations of factors that predicted IS events. Within model 1, we noted that shorter HD vintages and elevated ALP were selected as the most significant factors. While within model 2, we found recent access operation and serum RDW level as the two most significant factors without collinearity in a single regression model. Both combinations have adequate predictability for IS within future 1 month. Due to the natural rarity of IS [1, 7], there was limited sample size and it may be improper for us to add in more variables in light of it. However, based on our findings, we strongly suggest physicians to maintain a high degree of suspicion for infectious process facing symptoms of back pain, neurological deficit, unexplainable fever, weight loss, lethargy, or confusion in patients receiving maintenance HD, particularly if there has been an unexpected high ALP level, or inexplicable low RDW level following a recent access intervention in patients with relatively short HD vintage.

Intriguingly, we found that the study group had a shorter mean HD vintage before we included it as a matching variable. This could be partly explained by the fact that patients require a recent access operation to start hemodialysis, and a recent access operation is a significant factor for increased odds of IS. This significant relationship between shorter HD vintages and a recent access operation was indeed observed in our study, while adding HD vintage as an additional matching variable did not affect the significant relationship between a recent access operation and IS. In other words, factors besides recent access operation that relates to shorter HD vintage, such as the inexperience in self-care, may also contribute to this finding. Moreover, though longer HD vintages were reported to be related to declined nutritional status and increased mortality [25], our result showed a positive relationship between HD vintage and some markers of nutritional status, including serum albumin and hemoglobin levels (data not shown). These findings could be explained by the fact that all patients included were regularly followed up and treated in a tertiary medical center, and their nutritional status was closed monitored throughout the hemodialysis course. As a result, the better nutritional status over time may also explain the decreased odds of IS as the HD vintage gets longer. We believe two of the reasons discussed above both contributed to our findings because the HD vintages in the two study groups were still relatively shorter after stratification by recent access operation.

The limitations of our study include its observational design and the small effective sample size. Future studies with a greater sample size or a large prospective cohort design are warranted to confirm our findings.

In conclusion, we found that HD through catheter, history of degenerative spinal disease, immunosuppression status, or recent access operation, low hemoglobin or albumin levels, high RDW or ALP levels were associated with the subsequent diagnosis of IS in patients undergoing HD, facilitating early detection of the risk group. These findings also highlight the potential pathogenesis of blood access and malnutrition as a link between HD patients and IS, and whether improving these correctable factors could prevent HD patients from IS remains a subject for future studies.

## Conclusions

Based on this PSM study, we found that patients with shorter HD vintage, a recent access operation history, or HD through a tunneled cuffed catheter was significantly related to the development of IS after adjustment. In addition, lower hemoglobin, lower serum albumin, higher RDW, and higher ALP levels, were significantly linked to IS after adjustment. Concurrent presentations of the often unspecific IS symptoms with these risk factors in patients under maintenance HD should prompt clinical awareness towards IS, especially when unexpected high ALP levels, or inexplicable low RDW levels following a recent access intervention were found in patients with relatively short HD vintages.

## Additional file

Additional file 1: **Table S1.** Comparison of lab data in hemodialysis patients with and without infectious spondylitis (3 months before diagnosis). (DOCX 15 kb)

## Data Availability

The datasets used and/or analyzed during the current study are available from the corresponding author on reasonable request.

## Abbreviations

AC: Ante Cibum
ALP: Alkaline phosphatase
ALT: Alanine transaminase
BUN: Blood urea nitrogen
CI: Confidence interval
CKD: Chronic kidney disease
CRP: C-reactive protein
DM: Diabetes mellitus
ESR: Erythrocyte sedimentation rate
ESRD: End-stage renal disease
HD: Hemodialysis
hsCRP: High sensitivity C- reactive protein
IBM: Immersed Boundary Method
IS: Infectious spondylitis
MRI: Magnetic resonance imaging
OR: Odds ratio
PSM: Propensity score matching
RDW: Red blood cell volume distribution width
ROC: Receiver operating characteristic
SMD: Standardized mean difference
SPSS: Statistical Package for the Social Sciences
