# Supplementary material for: Identifying risk groups of infectious spondylitis in patients with end-stage renal disease under hemodialysis: a propensity score-matched case-control study
Source: BMC Nephrol. 2019 Aug 16;20:323. doi: 10.1186/s12882-019-1504-x (PMC6698043; doi:10.1186/s12882-019-1504-x)
Supplement: Supplementary file 1 — Table S1. Comparison of lab data in hemodialysis patients with and without infectious spondylitis (3 months before diagnosis). (DOCX 15 kb) [file 12882_2019_1504_MOESM1_ESM.docx]

Additional file 1: Table S1. Comparison of lab data in hemodialysis patients with and without infectious spondylitis (3 months before diagnosis)

| **Variables** | | **Patients with infectious spondylitis (n=12)** | **Patients without infectious spondylitis (n=48)** | ***p* value** |
| --- | --- | --- | --- | --- |
| Laboratory data (blood sample) | |  |  |  |
|  | Leukocyte count, 1000/μL | 7.52 ± 3.9 | 6.65 ± 2.3 | 0.31 |
|  | Hemoglobin, g/dL | 8.48 ± 1.3 | 9.79 ± 1.4 | 0.004 |
|  | RDW, % | 16.4 ± 1.7 | 14.0 ± 1.2 | <0.001 |
|  | Platelet Count, 1000/μL | 183 ± 75 | 202 ± 71 | 0.43 |
|  | BUN, mg/dL | 78.8 ± 37.5 | 64.3 ± 19.8 | 0.22 |
|  | Creatinine, mg/dL | 9.55 ± 4.52 | 9.66 ± 2.30 | 0.91 |
|  | Potassium, mEq/L | 4.80 ± 1.27 | 4.82 ± 0.81 | 0.93 |
|  | Calcium, mg/dL | 9.68 ± 1.63 | 9.70 ± 0.98 | 0.97 |
|  | Inorganic phosphorus, mg/dL | 5.25 ± 2.16 | 4.83 ± 1.62 | 0.45 |
|  | ALT , U/L | 22.8 ± 24.4 | 13.8 ± 5.8 | 0.38 |
|  | ALP, U/L | 156.3 ± 86.2 | 87.7 ± 49.3 | 0.002 |
|  | Albumin, g/dL | 3.47 ± 0.61 | 3.96 ± 0.47 | 0.005 |
|  | Glucose AC, mg/dL | 139 ± 54 | 132 ± 78 | 0.06 |
| RDW, red blood cell volume distribution width; ALT, alanine transaminase; ALP, alkaline phosphatase; BUN, blood urea nitrogen; AC, ante cibum | | | | |
| * No significant difference compared to data of 1 month before IS. | | | | |
